# The association between nurses’ burnout and objective care quality indicators: a cross-sectional survey in long-term care wards

**DOI:** 10.1186/s12912-021-00552-z

**Published:** 2021-02-23

**Authors:** Sameh Eltaybani, Noriko Yamamoto-Mitani, Ayako Ninomiya, Ayumi Igarashi

**Affiliations:** 1 Department of Gerontological Homecare and Long-term Care Nursing, The University of Tokyo, 7-3-1 Hongo, Bunkyo-Ku, Tokyo, 113-0033 Japan; 2 Department of Critical Care and Emergency Nursing, Alexandria University, Alexandria, Egypt; 3 The Dia Foundation for Research on Ageing Societies, Tokyo, Japan

**Keywords:** Burnout, professional, Cross-sectional studies, Japan, Long-term care, Quality improvement, Quality of health care

## Abstract

**Background:**

Worldwide, rather few studies have examined the association between burnout and care quality using objectively measured quality indicators, with most of the studies have relied on perceived quality outcomes. This study aimed to examine the association between staff nurses’ burnout and selected objective quality metrics in long-term care wards in Japan.

**Methods:**

This is a secondary analysis of a cross-sectional survey. Nurse managers and staff nurses working at randomly selected hospitals with long-term care wards—the equivalent of skilled nursing homes in Western countries— completed self-administered, anonymous questionnaires. The questionnaires collected data regarding care quality indicators, staff nurses’ burnout, and other confounders (e.g., ward size, participants’ years of experience, and patients’ conditions). All statistical analyses were conducted at the ward level. A multivariate regression analysis was used to examine associations between burnout and outcome indicators.

**Results:**

Data from 196 wards in 196 hospitals (196 nurse managers and 2473 staff nurses) across Japan were analyzed. Multivariate regression analysis showed that higher emotional exhaustion was associated with higher rates of pneumonia and pressure ulcers (*p*-value = .036 and .032, respectively), and that reduced personal accomplishment was associated with higher rates of tube feeding (*p*-value = .018). A larger ward size was also associated with low rates of pneumonia (regression coefficient = −.001, *p*-value = .019).

**Conclusions:**

Staff nurses’ burnout is a significant determinant of care quality in long-term care wards, implying that organizations that implement burnout reducing strategies may see greater benefits in patient outcomes. A large ward size was significantly associated with better care outcomes—specifically, low rates of pneumonia. Future research needs to determine feasible quality improvement measures in small-scale long-term care facilities, and to provide more comprehensive insights on ward-level variables that influence care quality in long-term care settings.

## Background

Burnout is a common, multifactorial phenomenon [1–4]. It is conceptualized as a prolonged response to chronic emotional and interpersonal stressors on the job, and is defined by the three dimensions of emotional exhaustion (EE), depersonalization (DP), and diminished sense of personal accomplishment (PA) [5]. The literature demonstrates that repercussions of burnout on care providers (e.g., attention deficits, anxiety, and insomnia) and organizations (e.g., high turnover, frequent employee absences, and frequent breaks) [6–8] can lead to poor-quality care (e.g., dissatisfaction with care, suboptimal patient care practices, and poor communication) and worsening patient safety (e.g., errors, adverse events, and safety perception) [9–13].

In long-term care (LTC) settings, nurses make up the largest proportion of the workforce [14], and they are exposed to a large number of stressors, including heavy workloads, time pressure, role conflict, role ambiguity, and physical tiredness [15, 16]. Besides, patients have complex needs, and a considerable number of them suffer from deterioration of cognitive and physical functions and impairment in the ability to communicate [3, 17]. Nurses in LTC settings, thus, are highly vulnerable to emotional strains, including burnout [18].

Worldwide, rather few studies have examined the association between burnout and care quality using objectively measured quality indicators [19], with most of the studies have relied on perceived quality outcomes, such as nurses’ perception of patient outcome [20, 21] and nurses’ rating of patient care [22]. The association between burnout and objective quality metrics is unclear, and therefore, we sought to investigate this association in LTC settings. Specifically, this study aimed to examine the association between staff nurses’ burnout and care quality in Japanese LTC wards using selected objective quality indicators, namely rates of pneumonia, rates of pressure ulcer, rates of using urinary catheters, and rates of using tube feeding. The principal research question was “Is objectively measured care quality in LTC wards related to staff nurses’ burnout?” Data reported in this manuscript are a part of a broader research project [23–25]. The current topic and results are distinct with no redundancy or duplication between the current study and any of the published studies from the same research project.

## Methods

### Design

This is a secondary analysis of a cross-sectional survey.

### Setting, sampling and participants

This study was conducted in LTC wards across Japan. In Japan, LTC wards are comparable to skilled nursing homes in Western countries [26]. They provide LTC for older adults with severe physical and cognitive problems. Patients are generally admitted from acute/subacute hospital wards after acute treatments, or from home due to exacerbation of their morbidities [26], and the average length of the stay is about 152 days [27]. Nurses and nurse aides are the primary care providers in Japanese LTC wards. Nurses’ role is basically the same as that in general hospital wards and involves a wide range of tasks, including organization of medication, maintaining physical cleanliness, changing diapers, assistance with bathing, providing medical assistance, checking vital signs, and nutrition management, as well as providing palliative care, dementia care, respiratory care, rehabilitation, and recreation [23, 28]. Nurse aides provide, in principle, life assistance activities for the patients; such as assistance with meal, cleanliness, bathing, and movement; and work environment maintenance activities to support nurses’ work; such as organizing nursing supplies and equipment, sorting out documents and slips, and bed making. The patient-to-nurse ratio and the patient-to-nurse aid ratio are both around 20:1 [29]. Out of 3767 hospitals with LTC wards in Japan, 2000 were randomly selected. The researchers sent a letter to the nursing directors of those hospitals explaining the study aim and asking whether they were willing to participate in the study. All nurse managers and staff nurses in the LTC wards of the hospitals that agreed to participate in the study were eligible for participation.

### Data collection

Nurse managers and staff nurses in the hospitals that agreed to participate in the study received self-administered, anonymous questionnaires. The nurse manager in each ward was responsible for distributing the questionnaires and then returning them to the researchers after completion via a pre-stamped envelope. Data were collected over three months (from September to November 2015).

### Measurements

Care quality was assessed using four outcome indicators: the rate of patients with pneumonia, the rate of patients with pressure ulcers (degree two or worse as defined by the National Pressure Ulcer Advisory Panel [30]), the rate of patients with urinary catheter, and the rate of patients on tube feeding. These indicators were adapted from previous research on care quality in LTC settings [31–33] and were derived from multiple interviews with experienced LTC nurses and researchers in Japan. Nurse managers reported these outcomes reflecting all patients in the ward on the day of completing the questionnaires. Rates were calculated by dividing the number of patients with each outcome by the total number of patients in the ward. Since the four indicators represent negative outcomes, higher rates indicate poor-quality care.

Staff nurses’ burnout was assessed using the Japanese Burnout Scale [34], which is based on the Maslach Burnout Inventory [35]. The Japanese burnout scale consists of 17 items under three sub-scales: EE (i.e., feelings of being overextended and depleted of one’s emotional and physical resources [5 items]), DP (i.e., a negative, callous, or excessively detached response to various aspects of the job [6 items]), and PA (i.e., feelings of incompetence and a lack of achievement and productivity at work [6 items]). Each item was rated on a 5-point Likert scale ranging from 1 (never experienced) to 5 (always experience). Higher scores on EE and DP and lower scores on PA indicate higher burnout. Therefore, the PA scores were reversed so that higher scores indicate higher burnout across the three subscales. Maslach and Jackson [35] stated that the three subscales represent separate, but related, aspects of burnout and are recommended not to be used collectively. In the current study, therefore, the summed score for each subscale was computed and used separately. In the current study, the Cronbach’s alpha coefficient was .860 for the whole scale and .832, .852, and .731 for the EE, DP, and PA subscales, respectively.

**Fig. 1:**
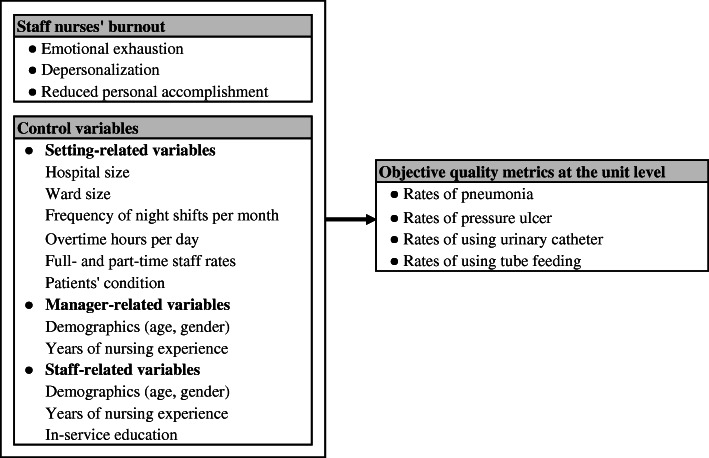
Conceptual framework of the study

Both burnout and care quality might be influenced by healthcare system-related factors (e.g., resources and management styles), care provider-related factors (e.g., socio-demographic variables and competency) and patient-related factors (e.g., patient illness) [36–40]. Therefore, for a robust examination of the association between burnout and care quality, including such confounding variables is vital. Nevertheless, including all possible confounders in a single study may not be feasible given their broadness and heterogeneity. For this research, three types of confounders were included: setting-, manager-, and staff-related variables (Fig. 1). Setting- and manager-related variables were collected from nurse managers, whereas staff-related variables were collected from staff nurses. Setting-related variables included hospital size, ward size, frequency of night shifts in the ward, rates of full-time and part-time staff in the ward, and patients’ condition. The latter was assessed by computing the proportion of patients in three levels of dependency based on the activities of daily living (ADL), with level 1 indicating the lowest dependency and level 3 indicating the highest. This is based on the sum of a seven-level score (0: independent to 6: completely dependent) in four functions (bed mobility, transfer, eating, and toilet use). Level 1 corresponds to scores of 0–10, Level 2 to 11–22, and Level 3 to 23–24 points. These levels are used in the case mix-based payment system in Japan [41]. Manager-related variables included managers’ age, gender, and years of nursing experience. Staff-related variables included staff nurses’ age, gender, years of nursing experience, and in-service education. The latter was assessed by the number of seminars each respondent has attended in the last three months whether inside or outside the hospital.

### Data analysis

All statistical analyses were conducted at the ward level. Data collected from staff nurses were aggregated at the ward level using the group mean. Following the univariate analysis, bivariate analysis using the Mann-Whitney U-test or Spearman’s correlation was performed to determine which variables were associated with each of the outcome indicators. Besides, multivariate linear regression analysis using the forced entry method was performed to examine associations between burnout and outcome indicators, controlling for variables with *p*-value < .2 with any of the outcome indicators in the bivariate analysis. Before running the regression analysis, the four outcome indicators were log-transformed to meet the linear regression assumptions, and all explanatory variables were mean-centered. Multicollinearity between potentially explanatory variables was also examined, and when a high correlation (i.e., *r* > .7) was found, the most clinically significant variable was selected to be included in the model. Two variables in the current study (ward size and number of seminars inside the hospital) had outliers (one value for each variable: 96 beds and 10.14 seminars per month, respectively). Therefore, all analyses involving these variables were conducted with and without including the cases with outlier values, and the results were compared (i.e., sensitivity analysis). All analyses were conducted using SPSS Statistics software version 23 for Windows. All reported *p*-values are two-tailed, and the .05 level was used for statistical significance.

### Ethical considerations

The Research Ethics Committee of the Graduate School of Medicine, The University of Tokyo, Japan, approved the current study (No. 10925). Along with the questionnaire, the researchers provided a letter stating the purpose and methods of the study, the voluntary nature of participation, and the confidentiality of responses. The researchers also stated that the completion and return of the questionnaires would be regarded as consent to participate. A permission to use the Japanese Burnout Scale [34] was obtained from the authors via e-mail.

## Results

**Fig. 2:**
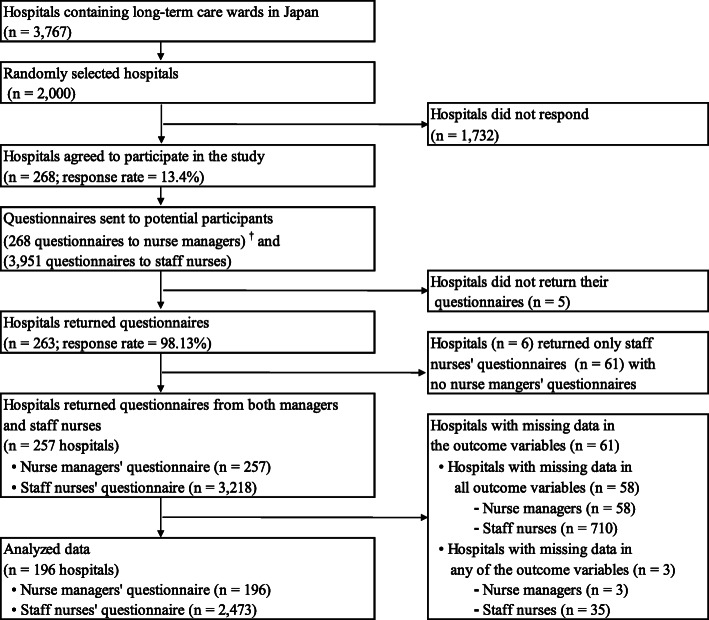
Sampling flowchart. ^†^One nurse manger per each ward and one ward per each hospital

A total of 268 hospitals participated in this study (response rate = 13.4%). Of those, 257 (95.9%) have returned questionnaires from both nurse managers (*n* = 257) and staff nurses (*n* = 3218). Questionnaires from 61 hospitals were excluded due to missing data in the outcome variables. Thus, questionnaires from 196 nurse managers and 2473 staff nurses from 196 LTC wards in 196 hospitals were included in the analysis (Fig. 2). The sensitivity analysis showed that outliers were not influential, and therefore, they were included in all analyses.

Table 1 summarizes the distributional characteristics of the study variables. The median (and interquartile range) hospital and ward sizes were 150 (102.3–199) and 48 (40–53), respectively. The median rates of patients with pneumonia, pressure ulcer, urinary catheter, and tube feeding were .03 (.0–.06), .05 (.02–.10), .17 (.11–.27), and .36 (.22–.54), respectively. Nurse managers were predominantly female (96.9%), their mean (± standard deviation) age was 51.85 (± 7.17) years, and their median years of nursing experience was 30 (22–33). About 95% of staff nurses were female, their mean age was 44.14 (± 10.87) years, and their mean years of nursing experience was 18.03 (± 10.99). The mean number of seminars in the last three months was 2.59 (± 2.23) seminars inside the hospital and .77 (± 1.36) seminars outside the hospital. Regarding staff nurses’ burnout, the mean score was 15.67 (± 4.80) for EE, 12.36 (± 4.83) for DP, and 22.63 (± 3.93) for reduced PA.

**Table 1:** Descriptive statistics of study variables

	Median [IQR], Mean ± SD, or n (%)
**1. Study settings-related data (*n*** **= 196)**	
Hospital size (number of beds)	150 [102.3–199]
Ward size (number of beds)	48 [40–53]
Average frequency of night shifts per month	5 [4–6]
Average overtime hours per day	.5 [.0–1.0]
Rate of full-time staff per bed	.49 ± .13
Rate of part-time staff per bed	.07 [.02–.15]
Proportion of patients with ADL level 1	.11 [.03–.23]
Proportion of patients with ADL level 2	.23 [.14–.33]
Proportion of patients with ADL level 3	.60 [.43–.79]
Care quality indicators	
Rates of patients with pneumonia	.03 [.0–.06]
Rates of patients with pressure ulcer	.05 [.02–.10]
Rates of patients with urinary catheter	.17 [.11–.27]
Rates of patients with tube feeding	.36 [.22–.54]
**2. Nurse managers-related data (*n*** **= 196)**	
Managers’ gender	
Female	190 (96.9%)
Male	5 (2.6%)
Managers’ age (in years)	51.85 ± 7.17
Managers’ years of nursing experience	30 [22–33]
**3. Staff nurses-related data (*n*** **= 2473)**	
Staff nurses’ gender	
Female	2335 (94.4%)
Male	135 (5.5%)
Staff nurses’ age (in years)	44.14 ± 10.87
Staff nurses’ years of nursing experience	18.03 ± 10.99
In-service education in the last three months	
Number of seminars inside the hospital	2.59 ± 2.23
Number of seminars outside the hospital	.77 ± 1.36
Burnout scores	
Emotional exhaustion	15.67 ± 4.80
Depersonalization	12.36 ± 4.83
Reduced personal accomplishment	22.63 ± 3.93

*IQR* Inter-quartile range, *SD* Standard deviation, *ADL* Activity of daily living

Percentages may not add to 100% due to missing values

The bivariate analysis (Table 2) showed that higher EE was associated with higher rates of pneumonia (*rho* = .152, *p*-value = .033) and pressure ulcer (*rho* = .175, *p*-value = .014). Reduced PA was associated with higher rates of urinary catheter (*rho* = .164, *p*-value = .021) and tube feeding (*rho* = .148, *p*-value = .039). Table 2 also shows that high proportions of patients with ADL level 3 were significantly associated with high rates of urinary catheter (*rho* = .392, *p*-value ≤.001) and tube feeding (*rho* = .525, *p*-value ≤.001). Rates of pneumonia were negatively related to staff nurses’ age (*rho* = −.189, *p*-value = .008) and their years of nursing experience (*rho* = −.172, *p*-value = .016).

**Table 2:** Bivariate analysis (n = 196) ^a^

	Rates of negative events (care outcomes) in the ward ^b^			
	Pneumonia	Pressure ulcer	Urinary catheter	Tube feeding
**1. Burnout** ^c^				
Emotional exhaustion	.152 **	.175 **	.079	−.083
Depersonalization	.140 *	.078	.086	−.060
Reduced personal accomplishment	−.069	−.059	.164 **	.148 **
**2. Setting-related variables**				
Hospital size	.038	−.056	−.016	−.014
Ward size	−.114*	.056	.056	.023
Average frequency of night shifts per month	.055	.029	.076	.081
Average overtime hours per day	.037	.048	−.057	−.117 *
Rate of full-time staff per bed	.102 *	−.009	.010	.024
Rate of part-time staff per bed	.045	−.036	−.034	.021
Patients’ condition				
Proportion of patients with ADL level 1	−.085	−.047	−.303 ***	−.400 ***
Proportion of patients with ADL level 2	.043	−.088	−.279 ***	−.467 ***
Proportion of patients with ADL level 3	.013	.117 *	.392 ***	.525 ***
**3. Manager-related variables**				
Managers’ gender				
Female	98.13	98.27	97.95	97.83
Male	93.20	87.90	99.80	104.30
Managers’ age	−.041	−.013	−.106	−.012
Managers’ years of nursing experience	−.023	−.040	−.101*	−.093*
**4. Staff-related variables** ^c^				
Percentage of female nurses in the ward	−.081	.023	.083	.079
Average of nurses’ age in the ward	−.189 **	−.090	−.104 *	−.048
Average of nurses’ years of experience	−.172**	−.091	−.079	−.041
In-service education ^c^				
Seminars inside the hospital	.085	−.058	−.054	.020
Seminars outside the hospital	.023	−.097	−.030	−.010

*ADL* Activity of daily living

^a^ All analyses were conducted at the unit level

^b^ Reported statistics are either correlation coefficients (Spearman’s rho) or mean ranks (for Mann-Whitney U-test)

^c^ Data were collected from staff nurses and aggregated at the unit level using the group mean

* *p*-value ≤ .2; ** *p*-value < .05; *** *p*-value < .001

Four variables were excluded from the multivariate analysis because of the multicollinearity: the DP sub-scale due to a high correlation with the EE sub-scale (*r* = .785; *p*-value ≤ .001); the average of staff nurses’ age in the ward due to a high correlation with the average of their years of nursing experience (*r* = .883; *p*-value ≤ .001); and the proportions of patients with ADL levels 1 and 2 due to high correlation with the proportion of patients with ADL level 3 (*r* = −.741; *p*-value ≤ .001 and *r* = −.715; *p*-value ≤ .001, respectively). The multivariate regression analysis (Table 3) showed that high EE was associated with high rates of pneumonia (regression coefficient [B] = .002, *p*-value = .036) and pressure ulcer (B = .002, *p*-value = .032). Reduced PA was associated with higher rates tube feeding (B = .008, *p*-value = .018). The regression analysis also showed that a larger ward size was associated with low rates of pneumonia (B = −.001, *p*-value = .019). Higher proportions of patients with ADL level 3 were associated with higher rates of urinary catheter (B = .072, *p*-value ≤ .001) and tube feeding (B = .123, *p*-value ≤ .001).

**Table 3:** Multivariate regression models (n = 196)

	Pneumonia rates	Pressure ulcer rates	Urinary catheter rates	Tube feeding rates
	B [95% CI]; ***p***-value	B [95% CI]; ***p***-value	B [95% CI]; ***p***-value	B [95% CI]; ***p***-value
Constant	.016 [.013–.018]; ≤ .001	.026 [.023–.030]; ≤ .001	.076 [.070–.082]; ≤ .001	.133 [.124–.141]; ≤ .001
Emotional exhaustion	.002 [.000–.003]; .036	.002 [.000–.004]; .032	.001 [−.002–.005]; .528	−.004 [−.009–.001]; .088
Reduced personal accomplishment	−.002 [−.004–.000]; .072	−.002 [−.004–.001]; .226	.005 [.000–.010]; .054	.008 [.001–.015]; .018
Ward size	−.001 [−.001–.000]; .019	.001 [.000–.001]; .597	−.001 [−.001–.001]; .598	.001 [−.001–.001]; .575
Number of overtime hours per day	−.001 [−.002–.002]; .820	−.001 [−.003–.002]; .601	.002 [−.003–.007]; .536	−.003 [−.010–.004]; .433
Rate of full-time nurses in the ward	.008 [−.016–.032]; .519	.006 [−.024–.036]; .691	.009 [−.047–.066]; .744	.029 [−.047–.105]; .455
Proportion of patients with ADL level 3	−.001 [−.011–.010]; .926	.012 [−.002–.025]; .087	.072 [.047–.098]; ≤ .001	.123 [.089–.157]; ≤ .001
Manager’s years of nursing experience	−.001 [−.001–.000]; .546	.001 [.000–.001]; .886	−.001 [−.001–.000]; .398	−.001 [−.002–.000]; .078
Average staff nurses’ experience in the ward	−.001 [−.001–.000]; .200	.001 [−.001–.001]; .925	−.001 [−.001–.001]; .916	−.001 [−.003–.001]; .349
*F*-test (p-value)	2.291 (*p* = .023)	1.131 (*p* = .345)	5.014 (*p* ≤ .001)	8.477 (*p* ≤ .001)
*R*^2^ (Adjusted *R*^2^)	.096 (.054)	.050 (.006)	.189 (.151)	.283 (.249)

*ADL* Activity of daily living

All dependent variables (y) are log-transformed (Log10[y + 1]), and all independent variables (x) are mean centered (x-mean)

## Discussion

The literature demonstrates the spiral down reciprocal association between burnout and care quality [42]. The current study is among the first to document the association between staff nurses’ burnout and objectively measured care quality in LTC settings. The findings suggest that interventions to manage staff burnout may positively impact not only staff but also patients [43], which highlight the significance of protecting and retaining the well-being and mental health of healthcare personnel [44]. The findings also revealed that different burnout sub-scales associate differently with different care outcomes, highlighting the distinct nature of the three burnout dimensions, and that using them collectively is not recommended [35]. Further, the current findings suggest that keeping a high proportion of experienced nurses in LTC settings may help to significantly improve patient outcomes.

In LTC settings, the high prevalence of burnout among nurses [3, 17, 45, 46] and its repercussions on care quality [46, 47] have been documented. For instance, higher burnout was associated with lower residential satisfaction [15], lower perceived quality-of-life [15], more depressive symptoms among residents [15], expressed emotions of criticism and hostility toward patients [48], and inadequate provision of comfort and support care [49]. Previous research also documented significant associations between hospital nurses’ burnout and negative patient outcomes, such as falls [20, 21] and nosocomial infection [19–21]. In other studies, however, there was no association between burnout and nosocomial infections [50] or pressure ulcer [11]. The current study confirmed that staff nurses’ burnout is a significant determinant of patient outcomes and that this association is not consistent in strength and significance across burnout sub-scales and the examined outcome indicators and may vary by the type of analysis. For instance, EE was associated with rates of pneumonia and pressure ulcers, but not with rates of urinary catheter or tube feedings in both bivariate and multivariate analyses. Conversely, reduced PA was associated only with rates of urinary catheter in the bivariate analysis and only with rates of tube feeding in the multivariate analysis. This is consistent with Salyers et al. [9], who reported that relationships between burnout and care quality differ by the type of burnout dimension, the unit of analysis, and source of quality rating.

Tawfik et al. [13] argued that objectively measuring care quality may not reliably identify certain events (e.g., near misses) and are difficult to connect to an individual provider (e.g., nurses) because of the team-based nature of caregiving. In contrast, subjective quality metrics might be more sensitive but more prone to bias (e.g., recall bias) [13]. Due to time and fund limitations, the current study relied on nurse managers’ reporting of patient outcomes instead of conducting direct observations. Nurse managers in Japanese LTC settings typically keep data regarding patient outcomes on the ward records, and therefore, the burden of reporting for the current study was minimal. By no means are the outcomes used in the current study exhaustive for evaluating care quality in LTC settings. Future research is required to establish the appropriate balance between the potential for bias with subjective quality measures and the insensitivity of objective measures [13].

The role of ward-level variables—such as infrastructure, staffing, high workload, high acuity of residents, presence of clinical education support, and high patient/carer ratio—in precipitating staff burnout and influencing care quality in LTC settings has been documented [13, 46, 51–53]. The bivariate analyses of the current study showed that the higher averages of staff nurses’ age and years of experience in the ward were associated with lower rates of pneumonia. This suggests that experienced nurses provide higher care quality than young, novice nurses, which may be explained by Benner’s [54] *Novice to Expert Theory*, implying that particular attention must be paid to young and novice nurses. The current study also showed that better care outcomes—precisely, low rates of patients with pneumonia—were related to a larger ward size. Previous research [55, 56] cited that strategies and innovation to enhance care quality may be challenging to implement and maintain in small-scale settings due to the lack of infrastructure, the lack of health information technology, limited resources, and the lack of adequate staff. These factors may, in part, explain the negative associations between the ward size and rates of pneumonia in the current study. Managers in small-scale settings, thus, need to pay more attention to care quality in their wards. This may include, but is not limited to, in-service training, reforming work patterns, and improvement of resources.

The outcome indicators examined in the current study are among the most reported in LTC settings worldwide. However, previous research on the association between burnout and these outcome indicators in LTC settings is scarce. The median rates of pneumonia (3%), pressure ulcers (5%), urinary catheters (17%), and tube feeding (36%) in the current study are comparable to those in the literature [57–62]. Although the current study is unable to determine whether burnout negatively affected care quality or whether negative outcomes caused burnout, findings may help to shed lights on potentially promising measures to improve care quality outcomes in LTC facilities. For instance, the current findings suggest that rates of pneumonia may be reduced by increasing the proportion of experienced nurses in the ward. Further, pneumonia prevention strategies [63, 64] need to be paid a particular attention by staff in small-size LTC facilities. Reducing nurses’ EE may be a promising strategy to help reduce rates of pneumonia and pressure ulcers in LTC facilities. Cimiotti et al. [19] argued that inadequate hand hygiene practices and lapses in other infection control procedures among nurses could be caused by cognitive detachment associated with high levels of burnout. Managers of LTC facilities with high rates of using urinary catheters and tube feeding need to pay a particular attention to nurses’ PA level. Interventions to minimize EE and enhance PA are cited in the literature and include individual-focused interventions (e.g., mindfulness, emotion regulation, stress management skills and communication skills training), organizational interventions (e.g., workload or schedule-rotation, stress management training program, debriefing sessions and a focus group) and combined interventions (e.g., stress management and resiliency training) [65, 66].

The current results are based on data derived from two distinct sources: nurse managers-reported data and staff nurses-reported data; and therefore, the possibility of personal bias is minimal. However, using a Likert scale to assess staff nurses’ burnout may be associated with a response bias. Besides, certain limitations of this study merit mention. First, this study provides only evidence of associations and is unable to determine a directionality or a causal relationship; future longitudinal studies are needed to clarify and confirm these relationships. Second, the low number of participating hospitals may raise questions regarding the generalizability of the findings; that is, only hospitals that were interested in providing high care quality might have agreed to participate in the study. The low number of participating hospitals also limited, to some extent, the inclusion of more predictors in the regression analysis. Third, the current results are based on a secondary analysis of a large-scale survey conducted primarily for other objectives. Therefore, of the numerous objective quality metrics in LTC settings [31–33, 67, 68], only four were examined in the current study. Some key care quality-related variables also were not included, such as the availability of audit systems, managerial style, professionals’ collaboration, and detailed patients’ characteristics. This limitation was evident in the low explanatory power of all regression models.

## Conclusions

This cross-sectional survey found that care quality in LTC settings is related to staff nurses’ burnout, and therefore, organizations that take proactive actions to reduce burnout may see greater benefits in terms of patient outcomes. The association between burnout and care quality are not consistent in strength and significance across burnout sub-scales and the outcome indicators. Rates of pneumonia and pressure ulcers in LTC facilities may be reduced by reducing nurses’ EE. Rates of using urinary catheters and tube feeding also may be reduced by enhancing nurses’ PA levels. Future research needs to determine feasible quality improvement interventions in small-scale LTC facilities and to provide more comprehensive insights on ward-level variables that influence care quality in LTC settings.

## Data Availability

The datasets of the current study are not publicly available due to containing information that could compromise the privacy of research settings and participants. Directors of participating hospitals and survey respondents were assured raw data would remain confidential and would not be shared.

## Abbreviations

ADL: Activity of daily living
DP: Depersonalization
EE: Emotional exhaustion
LTC: Long-term care
PA: Personal accomplishment
